# Prevalence, diagnostic evaluation, and disease associations of vector-borne pathogens in domestic dogs across Namibia: a multi-modal approach

**DOI:** 10.1186/s13071-025-06906-9

**Published:** 2025-07-10

**Authors:** Lourens de Villiers, Johan P. Schoeman, Barend L. Penzhorn, Umberto Molini, Mari de Villiers, S. Marcus Makgabo, Charles Byaruhanga, Nicola E. Collins, Peter N. Thompson, Samantha Zealand, Wilhelm H. Stoltsz, Ian J. M. Baines, Siegfried Khaiseb, Marinda C. Oosthuizen

**Affiliations:** 1 Department of Veterinary Tropical Diseases, Faculty of Veterinary Science, University of Pretoria, Private Bag X04, Onderstepoort, South Africa; 2 Department of Companion Animal Clinical Studies, Faculty of Health Sciences and Veterinary Medicine, University of Namibia, Neudamm Campus, Private Bag 13301, Windhoek, Namibia; 3 Department of Companion Animal Clinical Studies, Faculty of Veterinary Science, University of Pretoria, Private Bag X04, Onderstepoort, South Africa; 4 Istituto Zooprofilattico Sperimentale dell’Abruzzo e del Molise, 64100 Teramo, Italy; 5 Central Veterinary Laboratory, 24 Goethe Street, Private Bag 18137, Windhoek, Namibia; 6 Rhino Park Veterinary Clinic, 54 Rhino Street, PO Box 50533, Windhoek, Namibia; 7 Vaccine and Diagnostic Development Programme, Onderstepoort Veterinary Research, Agricultural Research Council, Private Bag X05 Onderstepoort, South Africa; 8 Department of Production Animal Studies, Faculty of Veterinary Science, University of Pretoria, Private Bag X04, Onderstepoort, South Africa; 9 SPCA Windhoek, 145 Robert Mugabe Avenue, PO Box 1495, Windhoek, Namibia; 10 Department of Paraclinical Studies, School of Veterinary Medicine, Faculty of Health Sciences and Veterinary Medicine, University of Namibia, Neudamm Campus, Private Bag 13301, Windhoek, Namibia

**Keywords:** Canine, Microscopy, Serology, Molecular, *Ehrlichia*, *Anaplasma*, *Hepatozoon*, *Babesia*

## Abstract

**Background:**

Due to limited documentation on vector-borne pathogens of companion animals in Namibia, a country-wide, multi-site field study was conducted to estimate the prevalence of these pathogens in domestic dogs.

**Methods:**

Samples of whole blood and serum from 375 dogs in 15 towns across eight regions were analysed. Vector-borne pathogens were screened by light microscopic examination of blood smears, point-of-care serology, and quantitative real-time polymerase chain reaction (qPCR). Haematology and serum biochemistry analyses were also performed.

**Results:**

Collectively, the SNAP^®^ 4Dx^®^ Plus Test provided 64% seropositive results, comprising *Ehrlichia* species (59%), *Anaplasma* species (45%), *Dirofilaria immitis* (2%), and *Borrelia burgdorferi* (< 1%). Altogether, prevalence as determined by probe-based qPCR assays was 54%, comprising *Ehrlichia canis* (27%), *Hepatozoon canis* (25%), *Anaplasma* species (13%), and *Babesia vogeli* (8%). Light microscopy yielded the least number of positives, indicating a collective positive result of only 11% in screening for *Ehrlichia*, *Anaplasma*, *Hepatozoon*, *Babesia*, and microfilaria species. On the whole, Kunene and Otjozondjupa regions showed the highest pathogen prevalence (75%), and the lowest was from Erongo region (38%), on qPCR testing. Significant associations between tick presence and infection by *E. canis* (*P* = 0.001), *Anaplasma* species (*P* = 0.006), and *B. vogeli* (*P* = 0.008) were demonstrated. Likewise, relevant associations between haemoparasite infection and variables of patient signalment, history, and various disease manifestations were shown. Finally, significant associations were found between pathogen infection and numerous clinical pathology abnormalities of the erythron, leukon, and thrombon, including thrombocytopenia (*P* = 0.022).

**Conclusions:**

Diagnostic modalities should be used contextually to test for canine pathogens, with due consideration of the limitations. Appropriate diagnostic testing such as qPCR, guided by relevant known associations with disease manifestation, should guide responsible treatment strategies and identify potential zoonotic risks in pets.

**Graphical Abstract:**

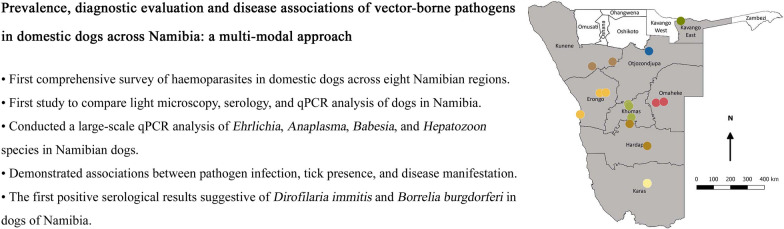

## Background

Information on the prevalence of vector-borne pathogens affecting companion animals in Namibia is limited [1]. Neglected economically important pathogens could significantly contribute to the disease burden of pets in the country [2]. The wide distribution of *Rhipicephalus sanguineus* sensu lato, the most prevalent tick infesting dogs in Namibia [3, 4], suggests that associated tick-borne parasites (including *Ehrlichia canis*, *Anaplasma platys*, *Babesia vogeli,* and *Hepatozoon canis*) [4, 5] may be present throughout the country.

*Ehrlichia canis* is the causative agent of canine monocytic ehrlichiosis, which presents with signs such as anorexia, lymphadenopathy, and mucosal haemorrhages and is characterised by thrombocytopenia as a key haematological feature [6]. Canine thrombocytotropic anaplasmosis, caused by *A. platys*, typically shows no or non-specific clinical signs, e.g. lethargy and pyrexia [7]. Zoonotic risks associated with *E. canis* [8–10] and *A. platys* [11, 12] infections have been reported. Babesiosis caused by *B. vogeli* usually leads to subclinical or mild infections but can manifest with lethargy, anorexia, and pyrexia [13]. Hepatozoonosis, caused by *H. canis*, generally results in mild disease with low parasitaemia, although more severe disease may occur in immunosuppressed dogs [14]. Diagnosing these pathogens can involve various methods, including light microscopy, serology, or molecular techniques [4, 15, 16].

A light microscopy study at Okahandja, central Namibia, reported *Babesia* (11%) and *Ehrlichia* species (14%) in dogs, as well as *Dirofilaria* (3%) and *Hepatozoon* species (2%) [17]. A small-scale study using light microscopy reported ehrlichiosis in various dog breeds in Windhoek, central Namibia [18]. Light microscopy has been used in neighbouring countries, such as the Republic of South Africa (RSA), to diagnose and quantify *Babesia* parasitaemia in dogs [19, 20].

Serology revealed exposure to *Ehrlichia* (40%) and *Anaplasma* species (23%) in rural dogs at Okahandja, central Namibia [4]. Additionally, seroprevalence of antibodies to *Ehrlichia* (54%) and *Babesia* species (69%) in dogs from central Namibia underscored the potential importance of these pathogens in the country [17, 21].

Molecular techniques are more sensitive than light microscopy or serology and effectively determine pathogen prevalence, as demonstrated in a large-scale study on South African dogs in which prevalence rates of *E. canis* (3%), *B. vogeli* (3%), and *B. rossi* (75%) were recorded [22]. Similarly, other molecular studies in South Africa have reported varying prevalence rates of *E. canis* (16% and 24%) [23, 24] and *A. platys* (19%) [24] in canine blood. In comparison, the effectiveness of molecular techniques was demonstrated through detection of *E. canis* (23%), *A. platys* (19%), *B. rossi* (0%), and *H. canis* (29%) in the blood of rural dogs from central Namibia [4]. Relatedly, the presence of *B. vogeli* was confirmed in blood samples collected from central Namibia by molecular techniques [5, 25].

The occurrence of vector-borne pathogens in dogs in the northern and southern regions of Namibia has not been documented, underscoring the need for a large-scale diagnostic study [1]. This would provide crucial insights into disease prevalence, vector presence, disease manifestation, and the distribution of economically significant pathogens across Namibia, enhancing our understanding of their clinical importance and informing effective treatment and preventive measures nationwide [1, 2]. Therefore, a multi-site field study was conducted to assess the current distribution of vector-borne pathogens in dogs throughout Namibia, using a multi-modal approach.

## Methods

### Study site and design

**Fig. 1:**
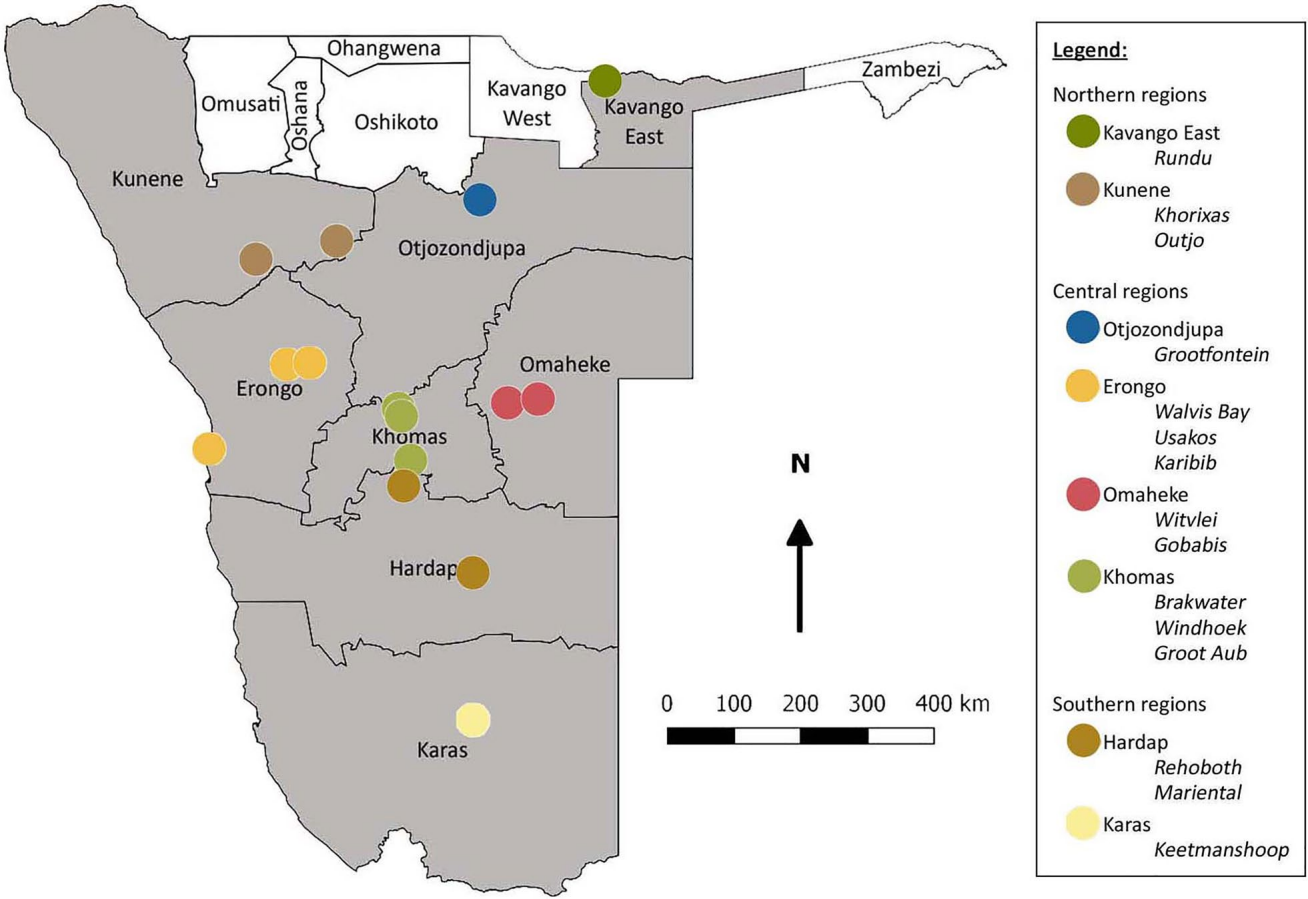
Canine sample origin sites across eight regions of Namibia

Whole blood and serum samples were collected from dogs in 15 towns in eight regions across northern, central, and southern Namibia (Fig. 1). With the exception of Windhoek, a major urban area in the Khomas region, all sites were rural, characterised by low human population density. Non-probability convenience sampling was integrated into routine diagnostic procedures for dogs receiving veterinary care at the Mobile Animal Clinic of the University of Namibia, from March to October 2022. Secondary analysis of stored samples and the use of patient metadata from medical records enabled investigation into the prevalence of pathogen infections in dogs. In total, 375 dogs were included in the study, with 43 to 50 dogs sampled per region.

### Sampling and processing

The sole inclusion criterion was dogs presented to the Mobile Animal Clinic, regardless of age, sex, breed, and health status, to avoid selection bias among healthy, clinically ill, or subclinically infected animals. Exclusion criteria encompassed sample contamination, inadequate quality or quantity of sample material, and samples linked to incomplete patient records. Clinical metadata from patient records were analysed, encompassing patient signalment, history, and clinical features. Particularly, key signalment information included patient sex, breed, and age. Relevant patient history comprised ectoparasite control, anorexia, and weight loss. Significant clinical features included the presence of ticks, pyrexia, lymphadenopathy, splenomegaly, bleeding diathesis, mucous membrane pallor, lethargy, and emaciation.

Peripheral capillary blood was collected using an ear prick, and a thin smear was prepared from the droplet. All smears were air-dried, fixed with Kyro-Quick Stain Fixative (Kyron Laboratories, Johannesburg, RSA) for 10 s, and stored for further processing. The smears were stained with a Kyro-Quick Romanowsky stain (Kyron Laboratories, Johannesburg, RSA) at standardised intervals, and stored at room temperature until microscopic examination. Typically, the stored blood slides were macroscopically evaluated for diagnostic adequacy, including a well-defined feathered edge and appropriate staining characteristics.

A needle and syringe suitable to patient size were used for venepuncture to collect central venous blood from either the jugular or cephalic vein. Thereafter, the whole blood was divided proportionally into serum and ethylenediamine tetra-acetic acid (EDTA) vials as needed. Packed cells were allowed to clot for 20–30 min at room temperature before cooled, while EDTA vials were immediately cooled to 4 °C. The serum was centrifuged at 8,000 rpm for 10 min before analysis. Due to logistical constraints, haematological and biochemical analyses, performed within 48 h of venepuncture, were limited to patients from the Khomas region. Aliquots of stored EDTA-anticoagulated blood and serum were kept in cryovials at −20 °C until further analysis.

### Haematology and biochemistry analyses

Haematological analyses were conducted using a CBC5R test kit (IDEXX Laboratories, Johannesburg, RSA) as per manufacturer guidelines, on an IDEXX LaserCyte Dx analyser equipped with software version 2.17 (IDEXX Laboratories, Johannesburg, RSA). Specifically, complete blood count indices, including red cell count (RCC), haematocrit (Hct), haemoglobin (Hb), mean corpuscular volume (MCV), mean corpuscular haemoglobin (MCH), mean corpuscular haemoglobin concentration (MCHC), red cell distribution width (RDW), reticulocytes, white cell count (WCC), differential WCC, and platelets were measured. Reference ranges for haematological results were applied on the basis of manufacturer guidelines. Biochemical analyses were performed in accordance with manufacturer guidelines, utilising an IDEXX CatalystOne analyser with software version 2.22 (IDEXX Laboratories, Johannesburg, RSA). More specifically, biomarkers assessed included total protein (TP), albumin (ALB), and globulin (GLOB). Reference ranges for biochemical results were determined according to manufacturer guidelines. Both haematological and biochemical results were managed using the IDEXX VetLab workstation (version 5.18). Reports were generated through the IDEXX VetConnect PLUS platform (IDEXX Laboratories, Johannesburg, RSA). Regular quality control checks were conducted on the LaserCyte Dx and CatalystOne analysers, following manufacturer guidelines, to ensure the accuracy and reliability of the test results.

### Light microscopy

Peripheral blood smears were examined by light microscopy. Initial screening at a low magnification (10× objective) across the entire feathered edge utilised a modified sideways battlement pattern to detect larger pathogens, such as microfilariae. Subsequently, the entire feathered edge was scrutinised at a higher magnification (100× objective with oil immersion). Any inclusions indicative of *Ehrlichia*, *Anaplasma*, *Babesia,* or *Hepatozoon* species were documented. Any other organisms of note were also recorded.

### Serology

The SNAP^®^ 4Dx^®^ Plus Test (IDEXX Laboratories, Johannesburg, RSA), an in vitro test, was utilised for enzyme-linked immunosorbent assay (ELISA) detection of *Dirofilaria immitis* antigen, antibodies to *Anaplasma phagocytophilum* or *A. platys*, antibodies to *E. canis* or *Ehrlichia ewingii,* and antibodies to *Borrelia burgdorferi*. Anticoagulated whole blood samples (*n* = 375) were allowed to thaw at room temperature for 30 min prior to analysis, following manufacturer guidelines. Colour development in the activation circle indicative of antigen or antibody presence was recorded.

### Extraction and molecular assays for pathogen detection

Deoxyribonucleic acid (DNA) was extracted from all blood samples using 200 µL of EDTA-anticoagulated whole blood using the PureLinkTM Genomic DNA Mini Kit (Thermo Fisher Scientific, Johannesburg, RSA), following manufacturer guidelines. The extracted DNA was eluted in 50 µL of elution buffer and stored at −20 °C until analysis.

Each DNA sample was subjected to four different quantitative real-time polymerase chain reaction (qPCR) assays for the detection of *Anaplasma* species (genus-specific assay) [26, 27], *E. canis* (species-specific assay) [28], *B. vogeli* and *B. rossi* (multiplex species-specific assay) [25], and *H. canis* (species-specific assay) [29]. A total of 10 µL of 2X TaqMan^®^ Universal Master Mix (Thermo Fisher Scientific, Johannesburg, RSA) and nuclease-free water quantum satis was used for all assays. The primers (Inqaba Biotechnical Industries, Pretoria, RSA) and probes (Integrated DNA Technologies, Whitehead Scientific, Cape Town, RSA) were as previously described [25–29], but the probes were designed and synthesised with modifications in several cases (Table 1). The primer and probe concentrations were optimised for each assay and the final concentrations used are shown in Table 1. A supplementary dataset on the optimisation of the qPCR assays is available.

**Table 1:** qPCR primer and probe concentrations used for screening of vector-borne pathogens in dogs

Target species	Primer/probe name	Sequence (5′-3′)	Final concentration (µM)	Final reaction volume (µL)
*Anaplasma* species [26, 27]	Ma16S_F	ACAGAAGAAGTCCCGGCAAA	0.8	20
	Ma16S_R	TTGCCCCCTCCGTATTACC	0.8	
	Ma16S_P	FAM-CCGTGCCAGCAGC-MGB-NFQ	0.15	
*E. canis* [28]	AnapEhrlichia_F	AGCYTAACACATGCAAGTCGAA	0.8	20
	AnapEhrlichia_R	TTACTCACCCGTCTGCCACTAA	0.8	
	E. canis_P	FAM-AGCCTCTGGCTATAGGA-MGB-NFQ	0.25	
*B. vogeli / B. rossi* multiplex [25]	BDog_F	TGTTGCAGTTAAAARGCTCGTAGTT	0.4	25
	BDog_R	AGTCTGCTTGAAACACTCTAATTTTCTC	0.4	
	Bvogeli_P	HEX-AGTTTGCCATTCGTTTGG-MGB-NFQ	0.25	
	BRossi_P	FAM-TGGCTTTTTGCCTTATTA-MGB-NFQ	0.25	
*H. canis* [29]	H. canis_F	GGCAGTGACGGTTAACGGGGG	0.4	20
	H. canis_R	GCACCAGACTTGCCCTCCAATTG	0.4	
	H. canis_P	FAM-CCGGAGAGGGAGCCTGAGAAACGG-BHQ1	0.25	

The qPCRs were performed using a Bio-Rad CFX96 thermocycler (Bio-Rad Laboratories, CA, USA). All assay cycling conditions consisted of an initial incubation at 50 °C for 2 min (one cycle), polymerase activation at 95 °C for 10 min (one cycle), followed by denaturation at 95 °C for 15 s and annealing/extension at 60 °C for 1 min (40 cycles). However, the *Babesia* multiplex assay had a modified annealing/extension step at 60 °C for 45 s (45 cycles) [25]. Data analysis was conducted using Bio-Rad CFX Maestro 1.1 (version 4.1) for Windows (Bio-Rad Laboratories, CA, USA). DNA extracted from known species-specific, mono-infected field samples served as positive controls, while molecular grade water was used as a negative control. The quality of positive control DNA was confirmed using a NanoDrop 2000 spectrophotometer (Thermo Fisher Scientific, Massachusetts, USA) to ensure reliability and accuracy.

### Statistical analysis

Descriptive statistics concerning patient signalment, history, and clinical features were reported as proportions. Pathogen prevalence in dogs per region, assessed using three different diagnostic techniques, was tabulated accordingly. The Shannon diversity index and Pielou evenness index (equitability) were computed to evaluate pathogen community diversity per region across various diagnostic methods. The Shannon index reflects richness, indicating differences in taxon numbers across regions, while equitability emphasises evenness, indicating differences in taxon abundance across regions. Fischer’s exact test was used to assess association between pathogen infections identified in whole blood by qPCR, co-infection, tick presence, patient signalment and history, and disease manifestation. All proportions were expressed as percentages, and statistical results were interpreted at a 5% level of significance. Statistical analyses were conducted using IBM SPSS Statistics (version 29.0) for Windows (IBM Corporation, NY, USA).

## Results

### Clinical presentation and disease manifestation

Patient signalment, history, and clinical features are indicated in Table 2. A supplementary dataset on the patient metadata is available. Most patients were male, mixed breed, and more than one year old. Endoparasite control was recorded in 36%, with 21% showing anorexia, 27% weight loss, and 62% tick infestation. Pyrexia and emaciation were each seen in 21%, lymphadenopathy in 32%, splenomegaly in 40%, and pale mucous membranes in 33%. Lethargy occurred in 3.2%, and few showed bleeding signs such as petechiae, ecchymoses, or epistaxis.

**Table 2:** Clinical metadata of canine patient signalment, history and clinical features

	Frequency	Proportion (%)
Signalment		
Sex		
Male	201	53.6
Female	174	46.4
Breed		
Mixed	340	90.7
Pure	35	9.3
Age		
< 1 year	98	26.1
> 1 year	277	73.9
History		
Ectoparasite control	133	35.5
Anorexia	77	20.5
Weight loss	100	26.7
Tick presence	233	62.1
Clinical features		
Pyrexia	77	20.5
Lymphadenopathy	121	32.2
Splenomegaly	127	39.9
Bleeding diathesis	17	4.5
Pallor	122	32.5
Lethargy	12	3.2
Emaciation	77	20.5

Total number of individuals (*N*) = 375 for each variable

### Vector-borne pathogen prevalence

A supplementary dataset on the diagnostic assessment of the vector-borne pathogens is available. Pathogens of interest were identified through light microscopy of blood smears (Table 3). *Ehrlichia* species were found in four regions, *Anaplasma* species only in Omaheke and Otjozondjupa, and *Babesia* species in all but Omaheke, Kavango, and Karas. *Hepatozoon* species were absent in Hardap and Karas. Protozoa were generally twice as common as rickettsial pathogens. Microfilaria were observed on smears from Erongo, Kavango, Otjozondjupa, and Karas. Pathogen diversity was generally high in richness and evenness, except in Kavango and Hardap; Karas showed no diversity. Equitability was highest (1.0) in Erongo and Omaheke, suggesting a lack of differences in abundance. *Mycoplasma*-like inclusions were also incidentally observed.

**Table 3:** Vector-borne pathogen prevalence as determined by light microscopy on blood smear samples from dogs across eight regions of Namibia

		Northern regions		Central regions				Southern regions	
	Total	Kavango	Kunene	Otjozondjupa	Erongo	Omaheke	Khomas	Hardap	Karas
Individuals (*N*)	375	47	47	47	47	47	50	47	43
Pathogen (%)									
All pathogens of interest	10.9	6.4	23.4	14.9	8.5	8.5	16.0	6.4	2.3
*Ehrlichia* species	2.4	0.0	4.3	0.0	0.0	4.3	6.0	4.3	0.0
*Anaplasma* species	0.5	0.0	0.0	2.1	0.0	2.1	0.0	0.0	0.0
*Babesia* species	3.7	0.0	12.8	2.1	2.1	0.0	10.0	2.1	0.0
*Hepatozoon* species	4.3	4.3	8.5	10.6	4.3	2.1	4.0	0.0	0.0
Microfilaria	1.1	2.1	0.0	2.1	2.1	0.0	0.0	0.0	2.3
Shannon diversity index	1.4	0.6	1.0	1.1	1.0	1.0	1.0	0.6	0.0
Equitability	0.9	0.9	0.9	0.8	1.0	1.0	0.9	0.9	n/a

Seroprevalence of antibodies to *Ehrlichia* and *Anaplasma* species is shown in Table 4. Kunene and Hardap showed the highest pathogen seroprevalence. *Ehrlichia canis*/*E. ewingii* had the highest collective seroprevalence (59%), peaking at 79% in Kunene and Hardap. *Anaplasma platys*/*A. phagocytophilum* seropositivity was found in all regions, being highest in Kunene (72%). *Dirofilaria immitis* was under 5% where seroprevalent, and absent in Erongo, Omaheke, and Karas. *Borrelia burgdorferi* antibodies were detected in one confirmed case from Omaheke. The SNAP^®^ 4Dx^®^ Plus Test does not detect *Babesia* antibodies. Overall, antibody-based diversity was high in richness and evenness; Erongo and Karas had equitability scores of 1.0, reflecting a more even diversity of taxa and correlated to the absence of *D. immitis* and *B. burgdorferi*.

**Table 4:** Vector-borne pathogen seroprevalence as determined by ELISA in whole blood samples from dogs across eight regions of Namibia

		Northern regions		Central regions				Southern regions	
	Total	Kavango	Kunene	Otjozondjupa	Erongo	Omaheke	Khomas	Hardap	Karas
Individuals (*N*)	375	47	47	47	47	47	50	47	43
Pathogen (%)									
All pathogens of interest	64.3	51.1	83.0	53.2	59.6	74.5	52.0	78.7	62.8
* E. canis*/*E. ewingii*	58.9	42.6	78.7	42.6	51.1	66.0	52.0	78.7	60.5
* A. platys*/*A. phagocytophilum*	44.8	31.9	72.3	46.8	31.9	51.1	22.0	68.1	34.9
* D. immitis*	1.9	2.1	4.3	2.1	0.0	0.0	2.0	4.3	0.0
* B. burgdorferi*	0.3	0.0	0.0	0.0	0.0	2.1	0.0	0.0	0.0
Shannon diversity index	0.8	0.8	0.8	0.8	0.7	0.8	0.7	0.8	0.7
Equitability	0.6	0.7	0.7	0.7	1.0	0.7	0.7	0.7	1.0

The qPCR screening revealed that pathogens of interest were prevalent across all regions (Table 5). Kunene and Otjozondjupa had the highest collective pathogen prevalence on qPCR (75%). *Ehrlichia canis* was found in all regions, being highest in Kunene (45%), and showed the highest total prevalence (27%). *Anaplasma* species were absent in Khomas but peaked in Kunene (28%). *Babesia vogeli* was present in all regions except Karas, being highest in Kunene and Otjozondjupa (13%), while *B. rossi* was not detected. *Hepatozoon canis* occurred in all regions, highest in Otjozondjupa (45%). Pathogen diversity was generally high in richness and evenness, though absence of *Babesia* in Karas lowered its diversity index. *Ehrlichia canis* (27%) and *H. canis* (25%) were collectively more prevalent than *Anaplasma* (13%) and *Babesia* species (8%).

**Table 5:** Vector-borne pathogen prevalence as determined by qPCR in whole blood samples from dogs across eight regions of Namibia

		Northern regions		Central regions				Southern regions	
	Total	Kavango	Kunene	Otjozondjupa	Erongo	Omaheke	Khomas	Hardap	Karas
Individuals (*N*)	375	47	47	47	47	47	50	47	43
Pathogen (%)									
All pathogens of interest	54.1	44.7	74.5	74.5	38.3	55.3	46.0	59.6	39.5
* E. canis*	27.2	10.6	44.7	34.0	19.2	23.4	18.0	36.2	32.6
* Anaplasma* species	12.5	14.9	27.7	14.9	12.8	14.9	0.0	10.6	4.7
* B. vogeli*	7.5	2.1	12.8	12.8	2.1	10.6	12.0	6.4	0.0
* B. rossi*	0.0	0.0	0.0	0.0	0.0	0.0	0.0	0.0	0.0
* H. canis*	24.8	23.4	34.0	44.7	14.9	23.4	32.0	17.0	7.0
Shannon diversity index	1.3	1.2	1.3	1.3	1.2	1.3	1.0	1.2	0.8
Equitability	0.9	0.9	0.9	0.9	0.9	1.0	0.9	0.9	0.7

### Comparison of diagnostic prevalence at regional level

**Fig. 2:**
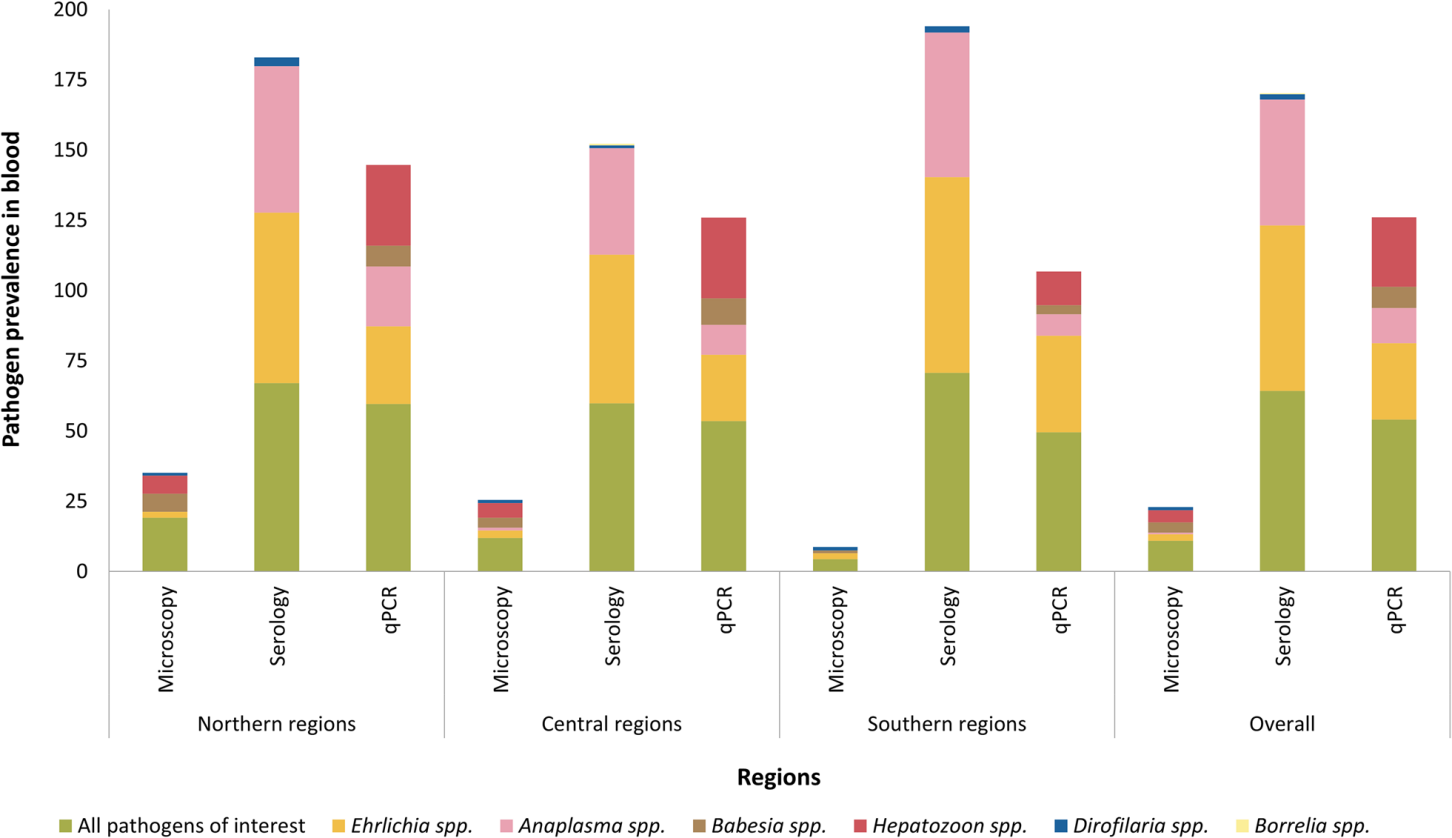
Macrogeographic variation in vector-borne pathogen prevalence as determined by various diagnostic modalities from whole blood in dogs across northern, central and southern regions of Namibia

Macrogeographic differences in diagnostic methods used to determine pathogen prevalence are evident (Fig. 2), where prevalence rates were averaged across northern, central, and southern regions. Collectively, serology revealed high exposure to *Ehrlichia* (59%) and *Anaplasma* (45%) species, while qPCR showed higher total detection (54%) than microscopy (11%).

Microscopy showed highest total pathogen prevalence in the north (19%), followed by central (12%) and southern (4%) regions. Specifically, *Ehrlichia* species were consistently detected (2–3%), *Anaplasma* only in central regions (1%), *Babesia* and *Hepatozoon* species were more common in northern (6%) and central (4–5%) regions than in the south, and microfilaria prevalence on smears were similar across regions (1%).

Altogether, seroprevalence was highest in the south (71%), with *Ehrlichia* exposure most common there (70%). *Anaplasma* seroprevalence was similar in north and south (52%) but lower in central (38%) regions. *Dirofilaria immitis* antigen detection was most frequent in the north (3%), while one sample from central Namibia was seropositive for *B. burgdorferi*.

Molecular detection mirrored microscopy, with prevalence highest in the north (60%), then central regions (54%) and the south (50%). *Ehrlichia canis* was most common in the south (34%), *Anaplasma* species in the north (21%), and *B. vogeli* and *H. canis* more frequently detected on qPCR in central and northern regions.

### Pathogen-tick-disease associations

Table 6 illustrates the significance of positive associations between patient metadata, including signalment, history, tick presence, and clinical signs, and pathogen infection identified by qPCR. *Ehrlichia canis* infection was significantly associated with male dogs (*P* = 0.010), mixed breeds (*P* = 0.028), age > 1 year (*P* = 0.025), anorexia (*P* = 0.046), weight loss (*P* = 0.013), lymphadenopathy (*P* < 0.001), splenomegaly (*P* < 0.001), bleeding diathesis (*P* = 0.023), pale mucous membranes (*P* = 0.004), emaciation (*P* = 0.002), and tick presence (*P* = 0.001). *Hepatozoon canis* infection was associated with mixed breeds (*P* = 0.004) and lymphadenopathy (*P* = 0.022) and negatively with ectoparasite control (*P* = 0.013). *Babesia vogeli* infection was linked to anorexia (*P* = 0.006), weight loss (*P* = 0.007), splenomegaly (*P* = 0.003), emaciation (*P* < 0.001), and tick presence (*P* = 0.008). *Anaplasma* species infection was associated only with tick presence (*P* = 0.006). No significant associations were found for pyrexia, lethargy, or other *Anaplasma*-related signalment or clinical signs.

**Table 6:** Associations of patient signalment, history, tick presence and clinical features with vector-borne pathogen infection as determined by qPCR in whole blood of dogs

	*E. canis*			*Anaplasma* species			*B. vogeli*			*H. canis*		
	*N*	%	*P*-value	*N*	%	*P*-value	*N*	%	*P*-value	*N*	%	*P*-value
Individuals (*N*)	102			47			28			93		
Signalment												
Sex			0.010^*^			0.160			0.167			0.339
Male	66	64.7		30	63.8		19	67.9		54	58.1	
Female	36	35.3		17	36.2		9	32.1		39	41.9	
Breed			0.028^*^			0.102			0.093			0.004^*^
Pure	4	3.9		1	2.1		0	0.0		2	2.2	
Mixed	98	96.1		46	97.9		28	100.0		91	97.8	
Age			0.025^*^			0.859			0.117			0.135
male < *1 year*	18	17.6		13	27.7		11	39.3		30	32.3	
female > *1 year*	84	82.4		34	72.3		17	60.7		63	67.7	
History												
Ectoparasite control	36	35.3	1.0	15	14.7	0.682	5	17.6	0.063	23	24.7	0.013^*^
Anorexia	28	27.5	0.046^*^	11	10.8	0.568	12	42.6	0.006^*^	18	19.4	0.882
Weight loss	37	36.3	0.013^*^	10	9.8	0.481	14	50.0	0.007^*^	28	30.1	0.418
Tick presence	77	75.5	0.001^*^	38	80.9	0.006^*^	24	85.7	0.008^*^	54	58.1	0.389
Clinical features												
Pyrexia	27	26.5	0.086	10	21.3	0.849	6	21.4	1.0	14	15.1	0.141
Lymphadenopathy	19	18.6	< 0.001^*^	13	27.7	0.509	7	25.0	0.529	21	22.6	0.022^*^
Splenomegaly	49	48.0	< 0.001^*^	16	34.0	1.0	17	60.7	0.003^*^	33	35.5	0.706
Bleeding diathesis	9	8.8	0.023^*^	0	0.0	0.145	2	7.1	0.368	4	4.3	1.0
Pallor	45	44.1	0.004^*^	13	27.7	0.508	14	50.0	0.057	29	31.2	0.799
Lethargy	4	3.9	0.742	1	2.1	1.0	2	7.1	0.223	2	2.1	0.738
Emaciation	32	31.4	0.002^*^	10	21.3	0.849	14	50.0	< 0.001^*^	25	26.9	0.103

^*^Fisher’s exact significance (two-sided): *P* < 0.05 = statistical significance

Table 7 demonstrates pertinent associations between abnormal counts from haematological and biochemical profiles, tick presence, and pathogen infections for Khomas. A supplementary dataset on the bloodwork analysis is available. Tick presence was significantly associated with increased lymphocyte counts (*P* = 0.010) and hypoalbuminaemia (*P* = 0.028) and commonly linked to decreased RCC, Hct, Hb, and thrombocytopenia. *Ehrlichia canis* infection was significantly associated with thrombocytopenia (*P* = 0.022) and frequent reductions in RCC, Hct, and Hb levels. All *B. vogeli*-infected dogs had decreased red blood cell (RBC) indices, and a negative association was observed with the Hb value (*P* = 0.022). Furthermore, *B. vogeli* was associated with reticulocytosis (*P* = 0.002), thrombocytopenia (*P* = 0.022) and hypoalbuminaemia (*P* < 0.001). *Hepatozoon canis* showed no significant bloodwork associations, though more than half had reduced RCC and Hct.

**Table 7:** Associations between haematology and serum biochemistry, tick presence and vector-borne pathogen infection as determined by qPCR in whole blood and serum of dogs from Khomas

	Tick presence			*E. canis*			*B. vogeli*			*H. canis*		
	*N*	%	*P*-value	*N*	%	*P*-value	*N*	%	*P*-value	*N*	%	*P*-value
Individuals (*N*)	26			9			6			16		
Haematology												
RCC	18	69.2	0.767	8	88.9	0.141	6	100.0	0.083	11	68.8	1.0
Hct	18	69.2	0.383	8	88.9	0.127	6	100.0	0.071	11	68.8	0.549
Hb	16	69.2	0.156	7	77.8	0.138	6	100.0	0.022^*^	8	50.0	1.0
MCV	1	3.8	1.0	0	0.0	1.0	0	0.0	1.0	1	6.3	0.542
MCH	1	3.8	1.0	0	0.0	1.0	0	0.0	1.0	1	6.3	0.542
MCHC	3	11.5	1.0	0	0.0	0.570	0	0.0	1.0	2	12.5	0.650
RDW	2	7.7	1.0	1	11.1	0.456	1	16.7	0.324	2	12.5	0.237
Reticulocytes	9	34.6	0.10	3	33.3	0.668	5	83.3	0.002^*^	4	25.0	1.0
WCC	8	30.8	0.327	1	11.1	0.425	2	33.3	0.621	4	25.0	1.0
Neutrophils	6	23.1	0.250	1	11.1	1.0	1	16.7	1.0	2	12.5	1.0
Lymphocytes	7	26.9	0.010^*^	1	11.1	1.0	2	33.3	0.192	3	18.8	0.666
Monocytes	5	19.2	0.420	2	22.2	0.595	2	33.3	0.192	3	18.8	0.666
Eosinophils	6	23.1	0.250	1	11.1	1.0	1	16.7	1.0	1	6.3	0.409
Basophils	7	26.9	0.050	1	11.1	1.0	0	0.0	0.572	4	25.0	0.249
Platelets	15	57.7	0.396	9	100.0	0.022^*^	6	100.0	0.022^*^	9	56.3	0.762
Serum biochemistry												
TP	11	42.3	0.242	3	33.3	1.0	3	50.0	0.396	2	12.5	0.053
ALB	11	42.3	0.028^*^	4	44.4	0.705	6	100.0	< 0.001^*^	4	25.0	1.0
GLOB	11	42.3	0.388	4	44.4	0.245	2	33.3	1.0	5	31.3	0.757

^*^Fisher’s exact significance (two-sided): *P* < 0.05 = statistical significance

## Discussion

This was the first comprehensive study to screen for vector-borne pathogens in dogs in the northern and southern areas of Namibia. Three diagnostic techniques were used to evaluate pathogen prevalence in dogs and disease manifestations were assessed. While a few small-scale studies have explored vector-borne pathogens in dogs from central Namibia, a comprehensive overview across the country has been lacking. This study aimed to estimate disease prevalence across eight regions, using a multi-modal approach, and establish associations with clinical disease manifestations. As a result, stakeholders can be informed on appropriate diagnostic methods and zoonotic risks, and, by extension, treatment and prognostication of vector-borne diseases in dogs. Significant associations were found between pathogen infection, tick presence, and disease manifestation. Comparatively, positive detection rates differed across serology, qPCR, and light microscopy for various pathogens – for example, *Ehrlichia* species demonstrated prevalence rates of 59%, 27%, and 2%, while *Anaplasma* species showed 45%, 13%, and 1%. The results of this study highlighted the need for careful consideration in the diagnostic approach to vector-borne pathogen infections in Namibia.

Most patients in this study were male, mixed-breed dogs older than one year. Significant positive associations were found between sex, breed, and age in *E. canis*-infected dogs, emphasising the relevance of these factors, although no breed predilection for German shepherd dogs was noted as in a previous study [18], possibly due to the low representation of purebreds. In contrast, the significant positive association of *H. canis* infection with breed likely reflects the high proportion of mixed-breed dogs, despite no known breed predisposition to hepatozoonosis. Rural, roaming dogs were generally at higher risk for infections, exacerbated by insufficient ectoparasite control, which increases susceptibility to vector-borne diseases.

Notably, only a quarter of *H. canis*-infected dogs had a history of recent ectoparasite treatment, and a negative association with infection suggests the importance of prophylaxis in preventing vector-borne diseases. History of clinical signs such as anorexia and weight loss were associated with *E. canis* and *B. vogeli* infections, in line with previous reports [6, 13], underscoring the need for thorough history-taking to identify clues to non-specific clinical signs. Furthermore, tick presence was significantly associated with *E. canis*, *Anaplasma* species, and *B. vogeli* infections, with over 60% of patients having ticks, similar to observations in a previous study [4], highlighting the ongoing need for community education on regular tick prevention. No association was found between tick presence and *H. canis*, possibly due to varied transmission routes. These findings indicate that the presence of ticks on a host should prompt diagnostic investigation for vector-borne diseases.

Pyrexia and lethargy were commonly observed but showed no significant associations with pathogen infections, cautioning against over-interpreting non-specific clinical signs without confirmatory diagnostics. Lymphadenopathy was strongly associated with *E. canis* infection and, unexpectedly, with *H. canis* infection, while splenomegaly showed significant positive associations with *E. canis* and *B. vogeli* infections. Notably, 61% of *B. vogeli*-infected cases presented with splenomegaly. Currently, this study is one of only few reporting a significant positive association between splenomegaly and *B. vogeli* infection. Bleeding tendencies were associated with *E. canis* infection but rarely observed, while mucous membrane pallor, in general, subject to being misinterpreted, was significantly associated only with *E. canis* infection. Emaciation was prevalent, particularly in infections with *E. canis* and *B. vogeli*, emphasising malnutrition and underlying disease concerns. Overall, the associations between pathogen infection with *E. canis* and *B. vogeli* and clinical signs were similar to previous reports [6, 13]. Plausibly, fewer associations between disease manifestation and infections by *Anaplasma* species and *H. canis* were observed because these pathogens typically cause subclinical or mild disease [7, 14].

Decreased RBC indices were notable in *E. canis*, *H. canis,* and *B. vogeli* infections, though no negative association with tick presence was found. A significant positive association between *B. vogeli* and reticulocytosis suggest a milder form of babesiosis, compared with more virulent species such as *B. rossi*, where regenerative responses are inadequate as a result of severe anaemia associated with bone marrow suppression [30]. The generally unremarkable WBC indices observed may be attributed to the variability in leukocyte response and severity of disease, caused by the pathogens investigated. Thrombocytopenia was common in *E. canis* and *B. vogeli* infections, as well as anaplasmosis, underscoring the importance of platelet count screening, as previously advised for these pathogen genera [6, 7, 31]. Hypoalbuminaemia was associated with tick presence and *B. vogeli* infection, suggesting inflammation-driven hypoalbuminaemia in the negative acute phase response, as also observed in cases of *B. rossi* infection [32]. No significant associations were found with hyperglobulinaemia.

This study highlighted the diagnostic value of qPCR over light microscopy in detecting vector-borne pathogens, similar to previous studies that compared microscopy and molecular techniques [15, 20]. *Ehrlichia* species were detected in 2% of dogs using light microscopy, compared with 27% by qPCR, highlighting that the majority of cases would be missed using microscopy alone. In contrast, previous studies in Namibia reported higher detection rates for *Ehrlichia* species using light microscopy (14%) [17] but similar rates with molecular testing (23%) [4]. These findings underscore the inconsistency of light microscopy compared with molecular techniques. Microscopic identification of rickettsial inclusions, such as *Ehrlichia* species morulae, can be challenging and time-consuming [6]*.* Notably, a large-scale study in South Africa reported a much lower prevalence of *E. canis* (3%) using molecular techniques [22], suggesting that *Ehrlichia* infections may comparatively be a more significant clinical concern in Namibia, likely due to factors such as geolocation and vector abundance. Other smaller-scale studies in South Africa showed variable molecular detection rates for *E. canis* [23, 24], with several aligning with findings in Namibia. Additionally, serological testing revealed substantial exposure to *Ehrlichia* species (59%). Previous studies in Namibia reported lower seroprevalence rates (40% [4] and 54% [21]) possibly due to regional differences, since those studies focused on central Namibia.

Regarding *Anaplasma* species, qPCR detected a prevalence of 13%, compared with just 1% by microscopy. Previous studies in Namibia (19%) [4] and South Africa (19%) [24] reported slightly higher detection rates using molecular techniques. The lower molecular detection of *Anaplasma* species in this study compared with *Ehrlichia* species suggests it may be of less clinical relevance in Namibia. However, serological tests showed a 45% seroprevalence for *Anaplasma* species. A prior Namibian study reported a lower seroprevalence (23%) [4], likely due to geolocation differences, since that study was limited to central Namibia.

This study detected *B. vogeli* in 8% of cases using qPCR and *Babesia* species in 4% through light microscopy. A previous study in central Namibia reported higher detection rates of *Babesia* species (11%) using microscopy [17]. Few studies have definitively identified and classified *B. vogeli* in central Namibia using molecular testing [5, 25]. The prevalence of *B. vogeli* in Namibia is relatively higher compared with a large-scale screening in South Africa, which found a 3% prevalence [22], suggesting that *B. vogeli* may play a more significant clinical role in Namibia. The absence of *B. rossi* in this study, as confirmed by molecular testing, aligns with previous findings [4]. This suggests that complicated babesiosis caused by *B. rossi* is unlikely to be a common disease manifestation in Namibia, unlike in South Africa, where *B. rossi* is found in high abundance (75%) in dogs [22] and is often associated with severe cases of canine babesiosis [32].

For *Hepatozoon* species, qPCR detected infections in 25% of dogs, while microscopy identified only 4%. These microscopy results are consistent with previous findings in Namibia, which reported a 2% detection rate for *Hepatozoon* species [17]. Unlike rickettsial species such as *Ehrlichia* and *Anaplasma*, gamont inclusions of *Hepatozoon* species are less prone to misidentification under microscopy. Similarly, the molecular findings from this study align with previous reports of *H. canis* (29%) in central Namibia [4]. These results highlighted that qPCR is significantly more effective in detecting haemoparasite infections, particularly for pathogens such as *E. canis*, *Anaplasma* species, *B. vogeli,* and *H. canis*, whose inclusions can be missed under light microscopy. However, the utility of light microscopy remains valued as a sensitive in-house diagnostic tool in the correct context, such as when applied for diagnosing infections such as *B. rossi* [19].

Previous studies in Namibia reported a low prevalence of microfilaria on blood smears (3%) [17], similar to the 1% found in this study. Comparatively, another study in central Namibia did not detect *D. immitis* in the blood or ticks of dogs using molecular techniques, nor any seroprevalence of heartworm [4]. Therefore, this study marks the first documented serological evidence which possibly suggests a low seroprevalence of heartworm in Namibia. However, previous studies have reported microfilaria such as *Acanthocheilonema dracunculoides* in Namibian dogs [33] and cross-reactivity with *Spirocerca lupi* using serological diagnostic test kits [34]. Therefore, additional investigation employing molecular techniques to confirm the serological findings presented here is justified. Additionally, while a previous study failed to detect *Borrelia* species seroprevalence [4], this study provides the first serological evidence which suggests exposure to *B. burgdorferi* in Namibian dogs and requires further molecular investigation to verify. These findings emphasise the limitations of relying on microscopy or serology alone and advocate for the use of qPCR to accurately detect and confirm canine vector-borne diseases.

Pathogen prevalence generally decreased from northern to southern Namibia based on both light microscopy and qPCR. This trend may have been influenced by a variety of factors, such as the environment (climate and biome), host and vector (population density and distribution), or socio-economic conditions [4, 35]. In contrast, collective seroprevalence based on serology was higher in the northern and southern regions, possibly owing to increased disease exposure in low-income, remote areas with poor housing conditions and limited access to veterinary services [17, 21]. Diagnostic methods varied in detecting pathogens across regions, with microscopy and qPCR showing greater differences for rickettsial species than protozoal species. *Babesia* and *Hepatozoon* species showed a north-to-south decline, aligning with the total pathogen detection trend. These protozoal species are easier to detect using microscopy and less prone to cross-reactivity in serological and molecular detection compared with rickettsial species such as *Ehrlichia* and *Anaplasma* species [7]. Specifically, *Ehrlichia* species were more common in the south, while *Anaplasma*, *Babesia,* and *Hepatozoon* were prevalent in the north. *Dirofilaria* species had a low seroprevalence across most regions, and *Borrelia* exposure was noted in a central region.

Several challenges and limitations were faced in this investigation. Non-probability sampling may have introduced bias, limiting the generalisability of prevalence estimates to all dogs. Metadata collection was subjective, relying on two operators, which could have influenced observations. Haematological and biochemical analyses were confined to Khomas owing to logistical constraints, limiting diversified regional representation. Holistically, the very low detection rates of light microscopy potentially limit its usefulness to evaluate prevalence rates [15]. Blood smear analysis focused solely on parasite detection, excluding morphological analysis. Additionally, no comparative serology for *Babesia* or *Hepatozoon* species was conducted, and qPCR testing for *D. immitis*, *B. burgdorferi,* and *Mycoplasma* species was omitted due to budget and logistical constraints. These limitations should be considered when interpreting the findings.

The study highlights several key recommendations for practitioners. A thorough understanding of clinical signs associated with specific pathogen infections is essential, but diagnosis and treatment decisions should not be based solely on clinical signs, without confirming the presence of the pathogen [7, 13, 14]. Expanding biomarker analysis beyond those used in this study is encouraged for future research [13]. Given the prevalence of *R. sanguineus* s.l. in Namibian dogs [3, 4], practitioners should routinely assess for ticks and investigate associated pathogens, even in subclinical patients. To the authors’ knowledge, in-house diagnosis in Namibia often relies on blood smear analysis and disease presentations, which can lead to misdiagnosis or inappropriate treatment in the absence of definitive pathogen confirmation. This underscores broader concerns about the emergence of novel pathogens, antimicrobial resistance, and zoonotic risks under the One Health paradigm [1, 2, 4], emphasising the need for access to effective diagnostic tools, such as qPCR. At the same time, without access to more reliable diagnostic methods like qPCR, practitioners should be aware that conventional techniques, such as light microscopy, may result in a lower detection rate for pathogens such as those described in this study [4, 15, 17].

The challenges of diagnosing ehrlichiosis in-house are heightened when treatment is based solely on suspicion or clinical signs, especially as molecular diagnostic tools, such as qPCR, become more accessible. With approximately one in four dogs in Namibia potentially having subclinical ehrlichiosis, there is a pressing need for responsible diagnostic practices to confirm infection before initiating antimicrobial treatment, which typically lasts at least a month [7]. Failure to confirm diagnoses risks contributing to antimicrobial resistance, particularly with the use of doxycycline [36]. While ehrlichiosis shows significant associations with disease manifestation, such as thrombocytopenia, many early findings are non-specific, underscoring the need for diagnostic confirmation [7]. In contrast, *Anaplasma* species infections are largely subclinical [7], and the debate over treating subclinical cases remains contentious. The authors recommend reserving advanced diagnostic testing, such as qPCR, for cases with evident clinical signs of anaplasmosis, and treatment decisions should be made carefully, considering the risks and benefits. Both practitioners and pet owners should remain cautious regarding the zoonotic risks associated with *E. canis* [8–10] and *A. platys* [11, 12] infections.

Relying solely on light microscopy for diagnosing *B. vogeli* can lead to missed cases, many of which are subclinical [13]. Given the strong associations reported in this study and previously [13] between *B. vogeli* infection and both non-specific signs, such as anorexia, weight loss, and emaciation, as well as specific signs, such as splenomegaly, practitioners should not dismiss babesiosis in Namibia as inconsequential. Thrombocytopenia as biomarker was shown to be significant in this study in cases of canine babesiosis. The authors recommend that the presence of *Babesia* species piroplasms on blood smear should prompt treatment. In cases of suspected *Babesia* species infection without smear confirmation, referral for qPCR testing is advised. For *H. canis*, typically subclinical [14], incidental findings on smear should be interpreted on the basis of parasitaemia and clinical signs, with treatment reserved for severe cases. Responsible antimicrobial use is key, since combination therapy may be considered [14]. Owners should be informed about the potential for relapse, particularly in cases of coinfection or immunocompromise, since treatment focuses on clinical improvement rather than complete parasitological cure [14].

The seropositive results for *D. immitis* and *B. burgdorferi* raise concerns about potential zoonotic risks [37, 38] and underscore the need for further investigations to molecularly confirm if caution is warranted for clinical cases presenting with signs suggestive of heartworm or *Borrelia* infection in Namibia.

## Conclusions

Associations between tick presence, pathogen infection, and disease manifestations in Namibian dogs were clearly demonstrated in this investigation. Furthermore, the study effectively highlighted the utility of different diagnostic techniques for pathogen detection in dogs. Indeed, the presence of ticks should prompt diagnostic investigation for vector-borne pathogens. Despite its limitations, light microscopy remains widely utilised for establishing a minimum database. The serology results underscore the distinction between pathogen exposure and active infection, necessitating careful interpretation of in-house serological findings. More importantly, the advocacy for routine use of molecular diagnostics, such as qPCR, to complement traditional methods is crucial. In conclusion, appropriate diagnostic testing, guided by relevant known associations with disease manifestation, should be the cornerstone in guiding responsible treatment strategies and identifying potential zoonotic risks in Namibian dogs.

## Data Availability

The datasets generated and/or analysed during the current study are available in the following repository: https://doi.org/10.25403/UPresearchdata.28350407.

## Abbreviations

ALB: Albumin
DNA: Deoxyribonucleic acid
EDTA: Ethylenediamine tetra-acetic acid
ELISA: Enzyme-linked immunosorbent assay
GLOB: Globulin
Hb: Haemoglobin
Hct: Haematocrit
MCH: Mean corpuscular haemoglobin
MCHC: Mean corpuscular haemoglobin concentration
MCV: Mean corpuscular volume
qPCR: Quantitative real-time polymerase chain reaction
RBC: Red blood cell
RCC: Red cell count
RDW: Red cell distribution width
RSA: Republic of South Africa
TP: Total protein
WCC: White cell count
